# Systematic Development of a Training Intervention to Support General Practitioners’ Very Brief Physical Activity Advice for Patients With Coronary Heart Disease: Formative Intervention Development Study From the OptiCor Project

**DOI:** 10.2196/89467

**Published:** 2026-10-22

**Authors:** Sabrina Kastaun, Sabrina Hoppe, Rik Crutzen, Daniel Kotz, Stefan Wilm, Alicia Prinz

**Affiliations:** 1 Institute of General Practice (ifam), Patient-Physician-Communication Research Unit, Centre for Health and Society (chs), Medical Faculty and University Hospital, Heinrich-Heine-University Düsseldorf, Moorenstr. 5, Düsseldorf, North Rhine-Westphalia, 40225, Germany, 49 0211 81 19527; 2 Department of Health Promotion, Care and Public Health Research Institute (CAPHRI), Maastricht University, Maastricht, Limburg, The Netherlands; 3 Institute of General Practice (ifam), Addiction Research and Clinical Epidemiology Unit, Centre for Health and Society (chs), Medical Faculty and University Hospital, Heinrich-Heine-University Düsseldorf, Düsseldorf, North Rhine-Westphalia, Germany; 4 Department of Family Medicine, Care and Public Health Research Institute (CAPHRI), Maastricht University, Maastricht, Limburg, The Netherlands

**Keywords:** primary health care, general practitioners, coronary heart disease, physical activity, brief intervention, behavior change techniques, formative research

## Abstract

**Background:**

Despite strong evidence supporting the benefits of regular physical activity (PA) for managing coronary heart disease (CHD), PA advice remains infrequent in general practice. Barriers include the limited familiarity of general practitioners (GPs) with current PA guidelines and their application to CHD patients, their insufficient skills for delivering very brief advice, and time constraints. To our knowledge, no structured, theory-informed training has been reported in Germany to support GPs in delivering very brief PA advice in routine CHD care.

**Objective:**

This intervention development study aimed to systematically develop and specify a training intervention to strengthen GPs’ delivery of very brief PA advice to patients with CHD in routine primary care.

**Methods:**

Guided by the behavior change wheel (BCW), we conducted a formative intervention development study informed by preceding empirical studies from the OptiCor project, including a cross-sectional survey, interviews, and focus groups with GPs and patients, alongside guideline recommendations and behavior change theory. These sources identified needs, barriers, and preferences related to PA advice. Findings were mapped onto COM-B (capability, opportunity, motivation-behavior) components to identify determinants of GP behavior and were specified using the theoretical domains framework (TDF). The BCW intervention function matrix informed discussions of suitable intervention functions and training components. Intervention components were specified using the behavior change technique (BCT) taxonomy, and GPs were involved throughout to ensure relevance, feasibility, and acceptability.

**Results:**

The development process resulted in a fully specified intervention: a structured, 3.5-hour, small-group training for GPs focusing on the very brief 3As method (ask/assess, advise, and assist). The training comprises theoretical input on PA and CHD, guided self-reflection and group discussions, moderated role-plays with simulated patients, experiential learning using PA stimuli, practical consultation materials, and short on-demand good practice videos. Outputs included a refined behavioral specification of GP-delivered PA advice, a COM-B/TDF-based behavioral diagnosis, BCT-specified training components, minor refinements after a preimplementation training rehearsal, and fidelity procedures to support standardized delivery.

**Conclusions:**

This study resulted in a fully specified GP training intervention to strengthen very brief PA advice in routine CHD care. By integrating empirical findings, guideline recommendations, behavior change theory, and GP and patient input, the intervention targets the determinants of GP advising behavior and is described transparently to support evaluation and replication. Following this development work, the training and evaluation procedures were examined in a pragmatic pilot cluster randomized controlled trial (cRCT), which has been reported separately, before proceeding to an ongoing full-scale pragmatic cRCT evaluating the effects on GP behavior.

## Introduction

### Background

Coronary heart disease (CHD) is one of the leading causes of morbidity and mortality worldwide [1] and is associated with reduced quality of life [2] and mental burden [3,4]. In Germany, the lifetime prevalence among individuals aged between 40 and 80 years is approximately 9% [5]. Beyond pharmacological treatment and revascularization procedures, the management of modifiable risk factors, such as smoking, unhealthy diet, and physical inactivity, plays a central role in secondary prevention.

While the role of physical activity (PA) in the care of patients with prevalent CHD was long overlooked by both health care providers and medical research, more recent systematic reviews have highlighted its effectiveness in managing this disease. Regular PA lowers cardiovascular mortality, recurrent cardiac events, and hospitalization rates, and it can improve quality of life in patients [6]. Nevertheless, approximately half of the affected population in Germany is physically inactive, a proportion higher than that in the general population [7].

The German national guideline on CHD care strongly recommends that health professionals (HPs), particularly general practitioners (GPs), advise and support patients in becoming more physically active [8]. GPs are well placed to do so, as they regularly see patients with CHD (eg, in the context of disease management programs) [9] and often have insight into their patients’ social context, families, barriers, and resources [10]. They are also regarded by patients as a trusted source of health information [9,11].

The World Health Organization (WHO) recommends brief interventions on PA as part of routine primary care for people with chronic diseases, considering them highly cost-effective [12-14]. Several methods exist to deliver brief or very brief advice on health behavior change in health care. Brief methods like the “5As” framework (ask, advise, assess/agree, assist, and arrange) and the more concise “3As” framework (ask/assess, advise, and assist) have proven to be practicable and effective in general practice  [9,10,15,16]. The “5As” framework typically requires around 10 to 15 minutes, whereas the more concise “3As” framework can generally be delivered within 5 to 10 minutes in routine consultations. Across all methods, providing concrete support (assist) is crucial, as it seems to be associated with the chance of behavior change [17]. A patient-centered approach to “assist” may include shared decision-making, individual goal setting, educational materials, or referrals to qualified regional or digital programs to support behavioral change [8,18].

Despite these recommendations, brief advice on PA is rarely given in German general practice [11,19,20]. A recently conducted population survey among individuals with CHD confirmed this, especially regarding concrete support [21]. A qualitative study with GPs further revealed that although GPs are aware of the health benefits of PA for patients with CHD, they rarely offer structured, personalized advice—often prioritizing advice on other behaviors like smoking cessation [22]. GPs report PA advice as time-consuming, ineffective, and frustrating, and express a need for structured processes and practical communication tools [22]. Barriers include time pressure, patient preference for medication, low motivation for lifestyle change, and few referral options [10]. A major issue is the lack of training in brief and effective behavior change counseling techniques [10]. Although international guidelines recommend such training for HPs [14,16,18], it is not yet part of undergraduate or postgraduate medical education in Germany, as is the case in many other countries [10].

Within our 5-year research program in health services research (OptiCor project) [23,24], we aim to systematically develop and evaluate an intervention to support GPs in delivering very brief PA advice in the routine care of patients with CHD [24]. This is done in line with the UK Medical Research Council framework for developing and evaluating complex interventions [25]. The decision to focus on training GPs to influence their advising behavior was based on both guideline recommendations and our prior success in developing and evaluating a brief GP training for smoking cessation advice [26]. While the target behavior in patients is different—encouraging engagement in PA instead of quitting tobacco use—there are important parallels. However, advising on PA also involves specific attitudes and barriers among GPs (eg, regarding its relevance for heart disease and how to address it effectively).

### Objective

This early report presents an intervention development study conducted within the OptiCor project. The study aimed to systematically develop and specify a training intervention to support GPs in delivering very brief PA advice to patients with CHD in routine primary care. Specifically, we aimed to identify behavioral determinants and implementation requirements for GP-delivered very brief PA advice; select intervention functions and behavior change techniques (BCTs) [27] to address these determinants; and describe the resulting training intervention and its fidelity procedures in sufficient detail to support subsequent evaluation, replication, and future adaptation.

The fully developed training constitutes the primary development output of this early report. Following the development work reported here, the training and evaluation procedures were examined in a pragmatic pilot cluster randomized controlled trial (cRCT) involving general practices and patients with CHD, which has been reported separately [28]. The present article focuses on the preceding formative development and specification of the training intervention.

## Methods

### Study Design and Reporting Framework

We conducted a formative intervention development study to produce a fully specified GP training intervention for subsequent pilot and full-scale evaluation.

The intervention development was guided by the behavior change wheel (BCW), incorporating the COM-B (capability, opportunity, motivation-behavior) model, and further informed by the theoretical domains framework (TDF) [29]. A multidisciplinary team comprising experts in psychology, sociology, general practice, health services research, and public health led the intervention development.

Reporting followed current recommendations for the development and reporting of complex interventions [25,30-33] and the Template for Intervention Description and Replication (TIDieR) checklist [34]. The completed TIDieR checklist is provided in Checklist 1.

### Setting and Overall Study Context

The intervention was developed within the 5-year OptiCor health services research program at the Institute of General Practice, Heinrich Heine University Düsseldorf [24]. GPs contributed in different roles: as advisers to interview-guide development and multiprofessional qualitative analysis during a preceding needs assessment (information is provided below); as participants in interviews and focus groups; as collaborators in developing the funding proposal, study design, training, training materials, and trainer manual; and as peer trainers in the preimplementation rehearsal and refinement. Their input shaped the brief, practice-oriented format, peer exchange, role-play, and practical resources. A simulated-patient trainer supported role-play development and rehearsal. Individuals living with CHD contributed as patient representatives or participants in the preceding needs assessment (information is provided below), informing the emphasis on individualized, motivational, and concrete support. One patient representative also co-developed the role-play case vignettes.

### Formative Evidence Base

The intervention development drew on multiple empirical sources from the broader OptiCor project as well as theoretical sources to inform the behavioral diagnosis and intervention design.

The formative sources included previous GP training research on smoking cessation advice (eg, [26,35]). A nationwide survey of individuals with self-reported CHD (N=1004) showed that only one-third had received PA advice covering all 3As (ask/assess, advise, and assist) since their most recent CHD-related event, with “assist” rarely implemented. Women and individuals with lower education reported even lower rates [21]. Qualitative interviews (n=12) and focus groups (n=6; 37 GPs in total) conducted between March and June 2023 explored barriers, including time constraints, frustration over perceived ineffectiveness, and patient safety concerns, and facilitators such as structured tools and communication strategies for PA advice [22]. GPs acknowledged the importance of PA but viewed it as secondary and often felt frustrated when advice was ignored. They called for training with peer exchange, evidence-based knowledge, practical tools, and efficient communication strategies [36]. In interviews (n=11) and focus groups (n=4; 22 participants in total), individuals with CHD described GPs’ advice as limited and expressed a need for individualized, motivational, and concrete PA support [37].

The formative evidence base also included guideline recommendations on PA advice in CHD care [8] and international recommendations on brief and very brief interventions for noncommunicable disease risk factors in primary care [12-14,18]. Further sources included behavior change frameworks and tools for intervention development, including the COM-B model, TDF, BCW, APEASE (affordability, practicability, effectiveness and cost-effectiveness, acceptability, side effects/safety, and equity) criteria, theory and techniques tool, and BCT Taxonomy v1 [27,29,38,39].

### Ethical Considerations

Ethics approval and informed consent were not required for the present formative intervention development study because no new participant-level data were collected or analyzed for this manuscript. The empirical studies that informed the intervention development were conducted as part of the broader OptiCor project under their respective ethics approvals and consent procedures, as reported elsewhere (eg, [21,22,24]).

### Development Process

Behavioral determinants of PA advising behavior were first mapped onto the COM-B model [29,38], which conceptualizes behavior as arising from capability (physical and psychological), opportunity (physical and social), and motivation (automatic and reflective). To achieve behavior change, all 3 must be sufficiently present; deficits in any component may therefore need to be targeted. To refine our behavioral diagnosis, we applied the TDF, which synthesizes 33 behavior change theories into 14 validated domains (eg, social influences and beliefs about capabilities).

Within the BCW, the COM-B model and TDF inform the selection of intervention functions—broad strategies such as education, training, modeling, persuasion, enablement, and environmental restructuring. Here, “training” describes the overall intervention format, which combines several BCW functions. Intervention development followed the 3-stage BCW process proposed by Michie et al [29], shown in Figure 1. Although presented sequentially, development was iterative and involved ongoing refinement. BCTs were specified using the established BCT taxonomy  [27].

**Figure 1.**
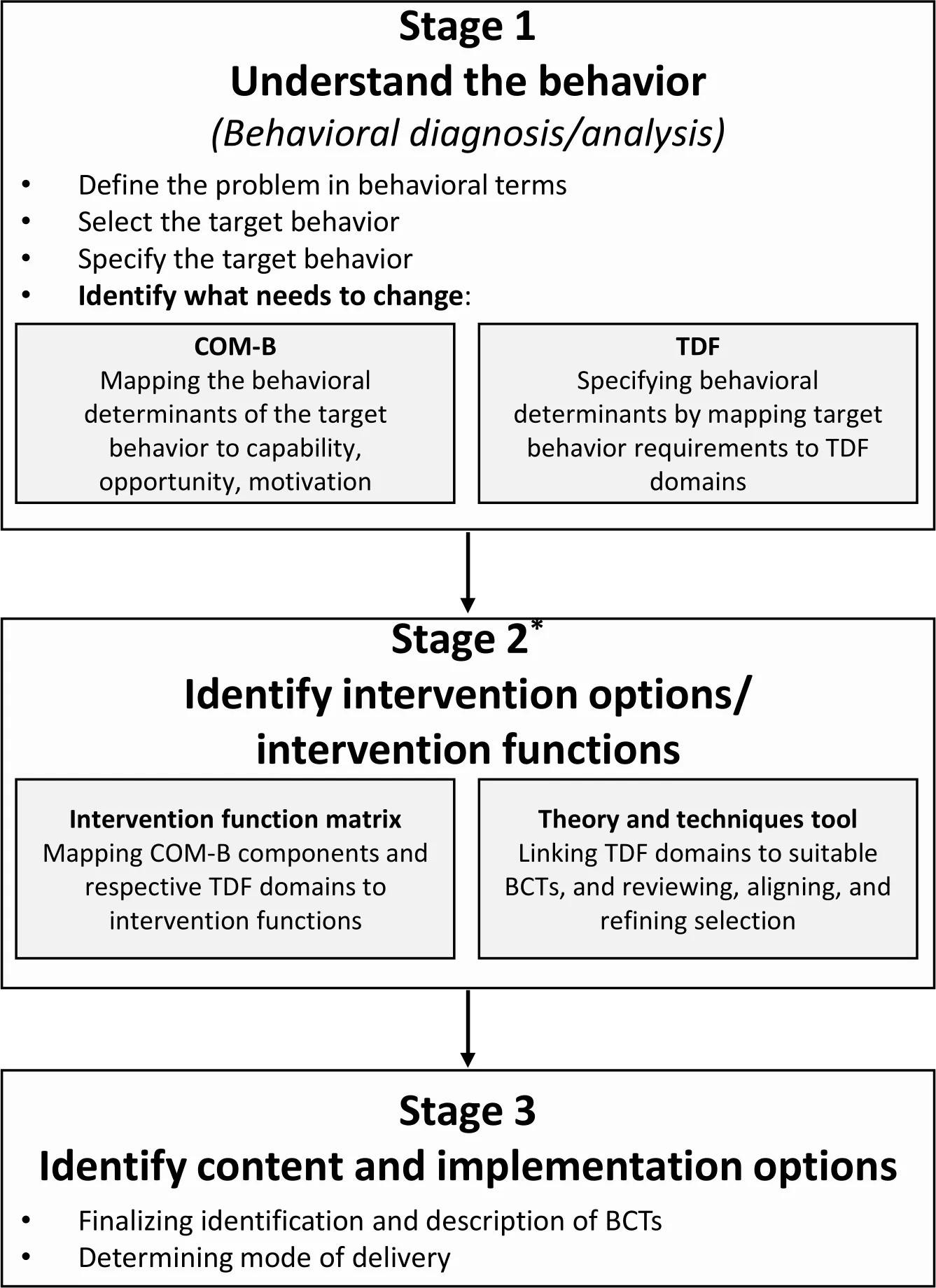
Stages of the behavior change wheel (BCW) intervention development process applied in the development of the OptiCor training intervention [29]. BCT: behavior change technique; COM-B: capability, opportunity, motivation-behavior; TDF: theoretical domains framework. *Policy categories (eg, legislation and regulation) were not considered, as the target behavior falls within the scope of implementation by individual general practitioners in routine care settings.

We selected the COM-B model and BCW because they integrate multiple behavior change theories, have proven feasibility in general practice  [26,40], and address both individual and contextual (eg, social and environmental) influences on behavior  [30]. They also provide a transparent, structured approach previously applied in interventions to improve lifestyle advice among HPs [41,42].

### Stage 1: Understand the Behavior

#### Definition, Selection, and Specification of the Target Behavior

The first steps of this stage were led by the project lead (SK) and a multidisciplinary team of collaborators (including GPs and public health, psychology, and health services researchers) prior to the funding application for the overall OptiCor study in June 2020. The target behavior was selected based on gaps between guideline recommendations and observed practice as outlined in the theoretical background above, as well as insights from routine GP care and previous studies [26,35].

We defined the target behavior as *opportunistic provision of very brief PA advice by GPs during consultations with patients with CHD*. Here, *opportunistic* means using suitable occasions in routine consultations, such as discussions of cardiovascular health, symptoms, or treatment, without scheduling a dedicated appointment. The intended standard was the concise 3As method (ask/assess, advise, and assist), chosen a priori for its brevity and feasibility and informed by prior smoking cessation research showing no advantage of the more complex 5As method [26,35].

After project launch and completion of the formative empirical studies, the target behavior was refined during a team workshop in early February 2024, applying criteria from Michie et al [29] (eg, impact, feasibility, spillover effects, and measurability). Spillover effects refer to unintended changes in other behaviors or patient groups resulting from the intervention (eg, improved advice on other lifestyle factors) [43]. Based on the literature [12] and our previous comparable studies [26], the behavior was rated as at least promising to very promising [29] in terms of impact, likelihood of achieving change, and feasibility of measuring change. However, evidence on potential spillover to other behaviors or patient groups was rated as uncertain due to insufficient evidence. The refined target behavior is presented in the Results section.

#### Identification of What Needs to Change

Findings from the formative evidence base were first synthesized in a structured study team workshop, producing a “big picture” of behavioral determinants mapped to COM-B components. A second workshop further refined the first training concept based on GPs’ content-related, didactic, and organizational requirements [36].

The detailed behavioral diagnosis, guided by “What needs to change to make the target behavior more likely?” [29], was initially developed by 2 researchers and later expanded using the TDF to specify COM-B components by detailing psychological, social, and environmental influences [29]. This enabled a comprehensive identification of barriers and enablers of the target behavior. This behavioral diagnosis was refined and validated in another study team workshop by the end of February 2024. The resulting behavioral requirements are presented in the Results section and in Table 1.

**Table 1.** Mapping of behavioral requirements to COM-B^a^/TDF^b^ domains, operationalized intervention functions, and core training components.

Behavioral requirements^c^ and TDF^d^ domain and COM-B component		Intervention functions^e^	Core training components and content examples
Psychological capability^f^			
Knowledge		Education	*Introductory lecture for theoretical input*. Definition of PA^g^, including everyday activities; evidence on its effects in chronic disease and health-related quality of life; WHO^h^ recommendations; evidence on the impact of GP^i^ advice; guideline recommendations; the 3As method; patient preferences and expectations; practical examples for everyday life; wearables for assessing and promoting PA; and reimbursement options for PA programs.
Cognitive and interpersonal skills		Training	*Moderated role-play sessions with peer feedback* to practice the 3As using conversation openers and simulated CHD^j^ cases.
Memory, attention, and decision processes		Training, environmental restructuring, enablement	*Introductory lecture*. *On-demand good-practice videos* after training showing standardized 3As-based PA advice and reinforcing knowledge. *Practical materials for GPs and patients*, including leaflets on recommended PA levels, regional PA program directories, 3As structure and PA conversation openers, the slides of the introductory lecture, and a visual reminder for GP offices. *Guided self-reflection and group discussions* to explore personal practice and barriers and reinforce theoretical knowledge.
Behavioral regulation		Education, training, modeling, enablement	*Introductory lecture*. *Practical materials offering PA opportunities and recommendations*. *Moderated role-play sessions with peer feedback*. *Guided self-reflection sessions and group discussions*. Collection of PA conversation openers.
Physical opportunity^k^			
Environmental context and resources		Training, environmental restructuring, enablement	*Introductory lecture*. The 3As method as a time-efficient approach that can be easily integrated into daily practice, including practical PA conversation openers. *Practical materials* offering PA opportunities and recommendations.
Social opportunity^l^			
Social influences		Environmental restructuring, modeling, enablement	*Guided self-reflection sessions and group discussions*. To exchange experiences, opinions, and attitudes on PA and patient-physician conversations about PA. *Introductory lecture* covering clinical guideline recommendations on PA advice for patients with CHD, its importance and effectiveness, and patient preferences regarding PA advice from GPs^m^.
Reflective motivation^n^			
Professional/social role and identity		Education, persuasion, modeling	*Introductory lecture* presenting current evidence on the effectiveness of PA in CHD; clinical guideline recommendations for PA advice; and its importance, effectiveness, and cost-effectiveness, along with patient preferences^m^. *Guided self-reflection sessions and group discussions*. Peer exchange to foster motivation, including everyday practice experiences, both positive and challenging (eg, barriers such as the GP’s own PA level), and a Monday question: “What will you change or do differently next Monday in your practice?” *Good practice videos available on-demand following the training*.
Beliefs about capabilities		Education, persuasion, modeling, enablement	*Introductory lecture*. *Practical materials offering PA opportunities and recommendations*. *Moderated role-play sessions with peer feedback*. *Guided self-reflection sessions and group discussions*. *Experiential learning through direct engagement using PA stimuli*. To raise PA awareness and provide a memorable training element, for example, by displaying simple training equipment (eg, resistance bands) and exemplary exercise instruction cards, and by talking about becoming physically active (eg, when having a break).
Optimism		Education, persuasion, modeling, enablement	*Introductory lecture*. *Practical materials offering PA opportunities and recommendations*. *Moderated role-play sessions with peer feedback*. *Guided self-reflection sessions and group discussions* (eg, on topics such as self-efficacy and frustration).
Beliefs about consequences		Education, persuasion, modeling	*Introductory lecture*. Patients are responsible for implementing PA; GPs play a key role in advising and supporting them. PA benefits patients with stable CHD, and the patient needs assessment indicated a clear desire for GP advice and support. *Moderated role-plays with peer feedback*.
Intentions		Education, persuasion, modeling, enablement	*Introductory lecture*. *Practical materials offering PA opportunities and recommendations*. *Moderated role-play sessions with peer feedback*. *Guided self-reflection sessions and group discussions*. Reminders integrated into everyday practice, such as patient-record prompts and adapted practice procedures.
Goals		Education, persuasion, modeling, enablement	*Introductory lecture*. *Practical materials offering PA opportunities and recommendations*. *Moderated role-play sessions with peer feedback*. *Guided self-reflection sessions and group discussions*.
Automatic motivation^o^			
Reinforcement		Training, environmental restructuring	*Introductory lecture*. *Practical materials offering PA opportunities and recommendations*. *Moderated role-play sessions with peer feedback*. *Guided self-reflection sessions and group discussions*. *Good practice videos available on-demand following the training*. *Experiential learning* through direct engagement using PA stimuli.
Emotion		Persuasion, modeling, enablement	*Introductory lecture*. *Practical materials offering PA opportunities and recommendations*. *Moderated role-play sessions with peer feedback*. *Guided self-reflection sessions and group discussions*. *Good practice videos available on-demand following the training*.

a COM-B: capability, opportunity, motivation-behaviour.

b TDF: theoretical domains framework.

c Physical capability was not targeted.

d TDF: An integrative framework comprising 14 domains that synthesize key theoretical constructs from relevant behavior change theories [29,43-45].

e All 9 behavior change wheel intervention functions were considered during the mapping process. The table presents the 6 functions retained and operationalized in the training.

f Requirements: Understanding the effectiveness of physical activity (PA) for the prevention of coronary heart disease. Knowledge of the effectiveness of providing very brief PA advice to patients. Knowledge and practical skills for delivering very brief advice using the 3As method: ask/assess, advise, and assist. Understanding guideline-based recommendations on PA intensity, frequency, and type.

g PA: physical activity.

h WHO: World Health Organization.

i GP: general practitioner.

j CHD: coronary heart disease.

k Requirements: Adequate time and an appropriate setting for brief PA discussions during consultations. Access to resources supporting brief PA advice, such as patient leaflets, a list of regional PA programs, relevant online resources, and recommended digital health applications.

l Requirements: Professional acceptance of providing advice on PA and discussing PA with patients. Social/professional/patient norms regarding PA advice.

m Based on current literature and our qualitative and quantitative needs assessment involving GPs and patients [21,22,24,36].

n Requirements: Belief that PA is effective in chronic disease management. Belief that very brief PA advice (3As) can be integrated efficiently into routine consultations and is a core responsibility of GPs. Belief that very brief GP advice can effectively promote PA (regardless of GPs’ own PA level). Belief that patients see their GP as a trusted source of health information and seek further guidance on PA. Belief that very brief PA advice is cost-effective. Belief that PA is accessible to everyone, regardless of individual characteristics or PA level, and can be integrated into daily life with minimal barriers. Perspective that patients are ultimately responsible for their health behavior change, while GPs play a key role in educating, motivating, and supporting them.

o Requirements: Reduced concerns about potential rejection from patients, the impact on the patient-physician relationship, or feelings of incompetence due to the GP’s own PA level. Confidence that effective PA advice does not require the GP to have an active lifestyle. Reduced perception that PA advice is time-consuming. Habitual use of the 3As as standard practice. Building resilience and motivation to engage in conversations about PA.

This behavioral diagnosis provided the empirical and theoretical basis for selecting intervention functions and designing the training components in the subsequent BCW stages.

### Stage 2: Identify Intervention Options

Building on the stage 1 behavioral diagnosis, we used the Intervention Function Matrix to map COM-B/TDF determinants to potentially suitable intervention functions [29]. The Theory and Techniques Tool [39], which links theoretical constructs to BCTs, was used to explore links between TDF domains and BCTs and identify or refine potential stage 3 intervention elements beyond those derived from our previous work [26].

All 9 BCW intervention functions were initially considered. Two team members mapped behavioral determinants to potentially relevant functions, which were then discussed by the multidisciplinary study team based on the formative evidence, guidelines, evidence on PA advice, and previous GP training research [26,35]. The APEASE criteria [29] informed these discussions pragmatically rather than through formal scoring. We considered plausibility, evidence, feasibility within the 3.5-hour format, available resources, acceptability, unintended consequences, and equity. Education, training, modeling, persuasion, enablement, and environmental restructuring were retained as the main operationalized functions. Restriction, coercion, and incentivization were not retained in the final mapping because no distinct corresponding intervention content was identified. Decisions were reached by consensus in March 2024 and are summarized in Table 1.

### Stage 3: Identify Content and Implementation Options

#### Overview

Stage 3 translated the behavioral diagnosis and selected intervention functions into concrete, deliverable training components and BCTs [27], mapped in Table 1 and Multimedia Appendix 1.

Qualitative findings on GPs’ preferences shaped the delivery mode: GPs expressed a need for peer exchange, self-reflection, evidence-based input, practical tools, and time-efficient communication strategies, with opportunities to practice through role-play [36]. Small, interactive groups, a trusting atmosphere, and experiential learning were preferred. Although format preferences varied between online and in-person delivery, accessibility, brevity, and compatibility with work and family informed group size, duration, and didactic format.

After the main training components had been specified, SH and AP systematically mapped all training elements to BCT Taxonomy v1 [27] using the training agenda, trainer manual, handouts, role-play structure, good practice videos, and consultation materials. SK reviewed the initial mapping, which was then discussed iteratively within the study team. To avoid overcoding, BCTs were assigned only when their active content was explicitly present and intended to contribute to changing GP advising behavior. Ambiguous cases were resolved by consensus. The final mapping was checked for consistency with the behavioral diagnosis, operationalized intervention functions, and training components. Examples are provided in Multimedia Appendix 1.

#### Preimplementation Training Rehearsal and Final Refinement

Feasibility and deliverability of the draft training were discussed in a workshop with experienced trainers from the research institute, including GPs, psychologists, and sociologists. The workshop addressed anticipated implementation challenges, such as potential reluctance among GPs to participate in role-play and concerns about PA safety in patients with CHD. The outcomes were incorporated into the trainer manual.

Before implementation in the subsequent pragmatic pilot cRCT, the complete training was rehearsed in a preimplementation training rehearsal and refinement session in August 2024. This session involved 3 GP peer trainers scheduled for initial training delivery, the scientific lead trainer, members of the study team, simulated patients, and the simulated-patient trainer. Its purpose was not to pilot the intervention or evaluation procedures, but to rehearse the full training flow under near-delivery conditions, including all materials, role-play procedures, simulated-patient encounters, and feedback sequences. Feedback from all participating groups was documented and informed final refinements, mainly streamlining lecture content on PA intensity in favor of everyday activity, clarifying the role-play feedback process, and reducing the difficulty level of 1 case vignette.

These refinement steps resulted in the training version described here and subsequently used in the pilot cRCT. The pilot cRCT and the resulting refinements for the ongoing full-scale pragmatic cRCT are reported separately [28].

#### Procedures to Support Fidelity and Trainer Standardization

Several procedures were used to support fidelity and trainer standardization: a detailed trainer manual, standardized materials, a small trainer pool, and on-site implementation support. Training delivery was restricted to 4 experienced GP peer trainers (2 regular and 2 back-up trainers) together with a scientific lead trainer experienced in GP training and behavior change intervention development.

The trainer manual specifies the training content, structure, timing, and core messages, along with role-play instructions, feedback rules, case vignettes, materials, and procedures for challenging situations during role-plays and discussions. Moreover, it provides background information on PA evidence and relevant studies to support trainers in responding to participants’ questions. Standardized slides, role-play vignettes, simulated-patient instructions, feedback prompts, handouts, practical materials, and the digital training portal are used across sessions. Consistency across sessions was supported through standardized case vignettes and instructions, initial training, and brief rebriefing before training sessions by an experienced simulated-patient trainer with experience in theater and film direction and theater education.

Delivery adherence was not monitored through formal empirical fidelity coding. However, one member of the scientific study team was present during the training session to support implementation, document substantial protocol deviations, and provide brief corrective guidance where necessary. Before the full-scale pragmatic cRCT, the trainers and the simulated-patient trainer received a 1-hour online refresher session to maintain consistency. Trainer manual and standardized material use, trainer briefing, study team supervision, and scheduled role-play block adoption were mandatory.

## Results

### Overview of Development Outputs

The formative development process resulted in a fully specified GP training intervention comprising a refined target behavior, a behavioral diagnosis, operationalized intervention functions, BCT-specified components, and refinements following a preimplementation rehearsal. The intervention is described using the TIDieR checklist (Checklist 1) [34].

### Stage 1 Outputs: Understanding the Behavior

#### Refined Target Behavior

The final target behavior was defined as initiating and delivering a very brief conversation on PA during routine consultations with patients with CHD whenever PA is clinically relevant and the consultation context allows it. The intended standard comprises the 3 core elements of the 3As model: ask/assess, which involves exploring current PA behavior, ideally capturing type, frequency, and intensity; advise, which involves emphasizing the importance of regular PA—encouraging those already active to maintain or increase PA and supporting inactive patients in becoming more active (eg, aerobic and strength activities); and assist, which involves providing tailored support, such as written PA recommendations, referrals, patient leaflets, regional program information, online or self-monitoring tools, follow-up, and joint consideration of opportunities for PA in everyday life or types of activities that are enjoyable and feasible.

GPs should routinely initiate these discussions, independent of patient characteristics or their own PA habits, within a patient-centered, shared–decision-making framework that respects patients’ individual preferences, resources, and prior PA experience.

The target behavior has been specified as “very brief” because the training is designed for routine GP consultations rather than dedicated lifestyle counseling sessions. The full 3As sequence is taught as the intended training standard, while the specific form of “assist” should be tailored to the individual patient’s needs, preferences, and resources, and the consultation context. In subsequent evaluation studies, the occurrence of a PA conversation and implementation of each 3As element have been assessed separately.

#### Behavioral Diagnosis and Requirements for Implementation

Based on the completed behavioral diagnosis, the following requirements define the conditions the intervention is explicitly designed to address, mapped to the COM-B domains (Table 1). Requirements related to *psychological capability* include understanding PA’s benefits in CHD, evidence for very brief PA advice, and mastery of all 3As steps, in addition to familiarity with guideline-based PA recommendations regarding type, frequency, and intensity. Requirements related to *physical opportunity* include time in consultations and access to materials such as leaflets, information on local PA programs, and recommended online resources or apps, or at least knowledge of where to find them. Requirements related to *social opportunity* include embedding PA advice as routine care, supported by professional norms, patient expectations, and a culture valuing prevention.

*Reflective motivation* includes belief in PA’s effectiveness and in very brief advice as feasible and integral to GP care; confidence in providing advice regardless of personal PA habits; appreciation of its cost-effectiveness and accessibility; and resilience to patient nonadherence. *Automatic motivation* involves overcoming concerns about rejection or harm to relationships, building habits of routinely giving very brief advice, and sustaining intrinsic motivation. No specific requirements have been identified for *physical capability*.

The behavioral diagnosis highlighted 3 central design implications. First, the training needs to address knowledge gaps regarding PA in CHD and current guideline recommendations. Second, it needs to strengthen communication skills for delivering the 3As model within limited consultation time. Third, it needs to address motivational and emotional barriers among GPs, including doubts about effectiveness, frustration after previous unsuccessful advice, concerns about whether PA can be addressed safely in CHD care, and concerns that advice might be rejected or harm the consultation relationship.

Table 1 presents the mapping of behavioral requirements to COM-B/TDF domains, operationalized intervention functions, and corresponding training components.

### Stage 2 Output: Selected Intervention Functions

The main operationalized intervention functions were education, training, modeling, persuasion, enablement, and environmental restructuring. These were translated into concrete training components, including theoretical input, guided self-reflection, peer discussion, role-play, feedback, good practice examples, practical consultation materials, prompts, and digital resources.

### Stage 3 Outputs: Training Content and Implementation

#### Overview

All core training components were specified using the BCT Taxonomy v1. Examples of BCTs operationalized in the training include instruction on how to perform the behavior, demonstration of the behavior, behavioral practice/rehearsal, feedback on the behavior, information about health consequences, prompts/cues, adding objects to the environment, and social support. Multimedia Appendix 1 provides examples of how selected BCTs were operationalized in the training.

#### Preimplementation Training Rehearsal and Final Refinements

The preimplementation training rehearsal indicated that the training was deliverable within the planned 3.5-hour format. Feedback informed three refinements: (1) streamlining content on PA intensity in favor of everyday activity, (2) clarifying the role-play feedback process, and (3) reducing the difficulty of 1 case vignette. No core component was removed or substantially changed. Training delivery and study procedures were subsequently examined in the pilot cRCT [28].

#### Training Intervention

The postrehearsal training intervention subsequently used in the pilot cRCT [28] comprises six core components: (1) an introductory lecture on theoretical foundations; (2) guided self-reflection sessions and group discussions; (3) moderated role-play sessions with peer feedback; (4) experiential learning through direct engagement with PA stimuli; (5) practical materials offering PA recommendations for GPs and patients; and (6) good practice videos available on demand after the session. Figure 2 illustrates how these 6 components map onto the COM-B model components influencing GP advising behavior.

**Figure 2.**
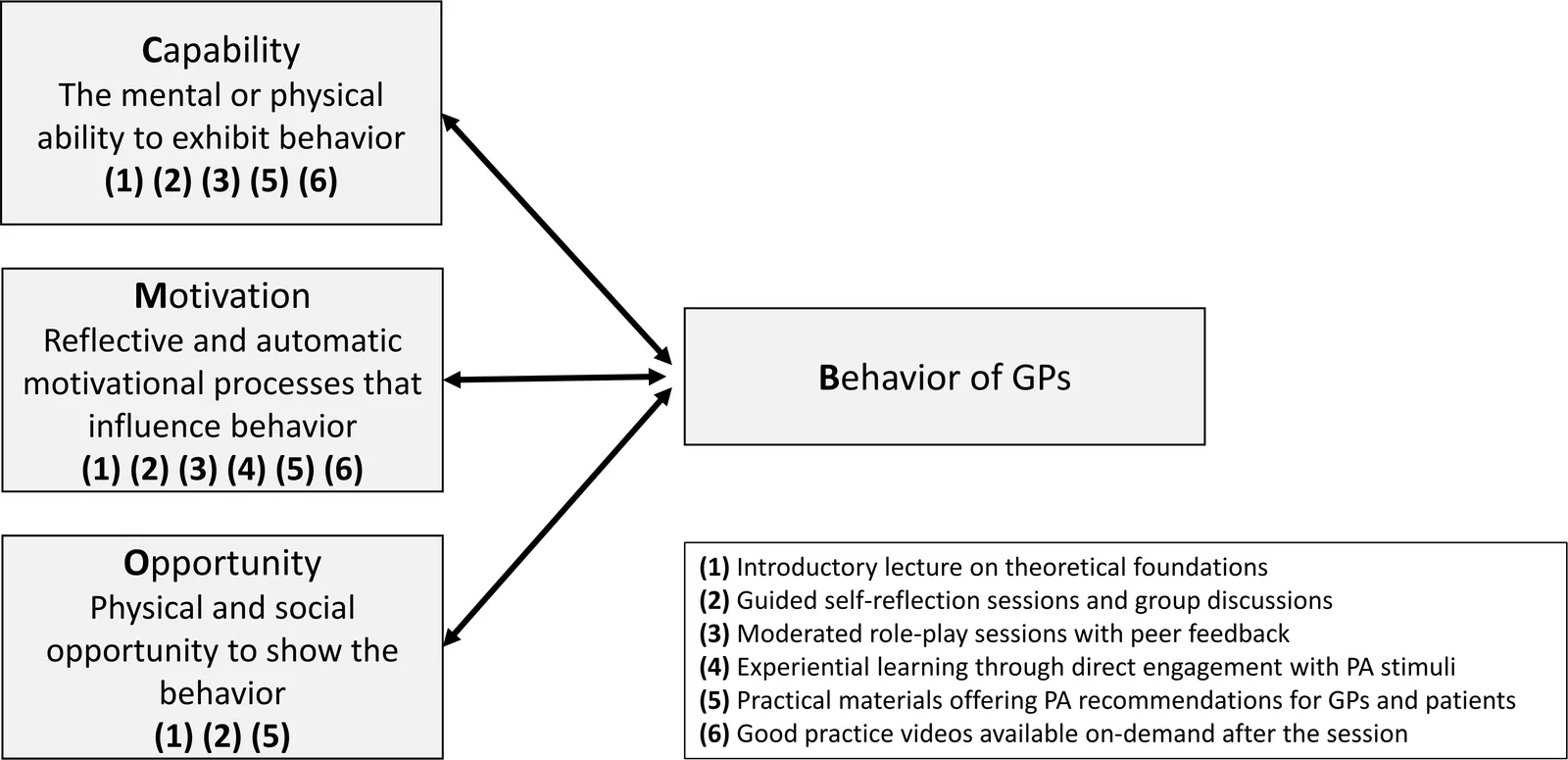
Mapping of the 6 core components of the developed GP training onto COM-B model components influencing GP advising behavior. COM-B: capability, opportunity, motivation-behavior; GP: general practitioner; PA: physical activity.

### Fidelity and Trainer Standardization

#### Overview

The final fidelity approach comprises a restricted trainer pool, detailed manual, standardized materials, simulated-patient preparation, and on-site implementation support.

#### Trainer Manual

A comprehensive manual (version 7; May 11, 2024; 36 pages; German language) specifies the organization, structure, timing, didactic approach, and theoretical and practical content of the training. It also includes background information on study evidence, instructions for facilitating role-plays, patient case vignettes, all handouts, and a “frequently asked questions” section addressing potential challenges (eg, GPs’ reluctance to participate in role-plays and participants questioning the evidence). Trainers receive the manual and printed presentation slides before the session. An English‐translated table of contents is provided in Multimedia Appendix 2.

#### Organizational Structure

Regarding group size and duration, small-group sessions run for 3.5 hours, with a maximum of 12 GPs per group (if reached). A training session can be scheduled on Wednesday or Friday afternoons and Saturday mornings, aligning with lighter GP practice loads or home-visit days in Germany. The trainer team consists of an experienced tandem of a GP peer trainer and a scientific lead (eg, psychologist), with 1‐2 study staff present for logistical support. Training takes place in small classrooms/seminar rooms—in our case, at Heinrich-Heine University Düsseldorf. The room is divided into two areas: (1) a lecture/discussion space with chairs, tables, writing materials, and slides for theoretical input and group reflection, and (2) a circle of chairs for role-plays, with large posters displaying rules for peer feedback and the 3As method as visual aids. Flipcharts are used to document participants’ comments during discussions.

Regarding organizational materials and catering, participants are provided at the entrance with name tags, attendance lists for continuing medical education (CME) credits, evaluation forms, and travel reimbursement forms. CME credits and travel reimbursement are administrative arrangements, not intervention components or BCW incentivization. A light catering service is available, offering beverages and snacks such as sandwiches, biscuits, and fruit. An overview of the training schedule is provided in Multimedia Appendix 3 (English translation).

### Description of Core Intervention Components and Their Development

#### Introductory Lecture

The session opens with brief introductions, during which participating GPs share their expectations for the training. This is followed by an introductory lecture given by the scientific lead, providing an overview of CHD epidemiology, prevalence, morbidity, mortality, and relevant risk factors.

The introductory lecture on theoretical foundations is divided into 2 parts, with a discussion and self-reflection in between. Part 1 addresses the general and CHD-specific health benefits of PA, referencing WHO guidelines and promoting a broad understanding that includes everyday activities. Current evidence on the effects of regular PA on chronic conditions like CHD is presented, emphasizing improved quality of life and effects comparable with pharmacological treatments. This part also aims to reinforce the core message that “every step/every minute counts.”

Additional topics include patient preferences for PA advice; guideline-based recommendations on the type, intensity, and duration of PA; practical examples; use of wearables (eg, smartwatches); reimbursement options for PA programs; and patients’ expectations regarding GP advice. Common concerns, including patients’ worries about overexertion, are addressed cautiously and pragmatically by emphasizing that PA is safe and guideline-recommended in stable CHD without overemphasizing risk. GPs are encouraged to recommend a gradual start, discuss warning symptoms such as dizziness or nausea, arrange follow-up, and consider referral to cardiac rehabilitation or cardiac sports groups where appropriate.

Following part 1 of the lecture, participants discuss key points and questions and engage in guided self-reflection on their own experiences delivering PA advice in routine care, including successful approaches and common challenges.

Part 2 presents evidence on the effectiveness and cost-effectiveness of very brief PA advice in primary care and introduces the 3As model as a concise framework. Trainers demonstrate each step, while participants contribute good-practice examples of conversation openers and personalized recommendations. GPs are reminded that patients remain responsible for behavior change, and GPs play a key role in initiating and supporting these conversations. A detailed lecture outline is provided in Multimedia Appendix 4.

The brief introduction, both lecture parts, and the intermediate discussion take about 60 minutes, with slight variations depending on participant questions and engagement. At the end of the 3.5-hour training, the expectations shared at the beginning are revisited.

#### Guided Self-Reflection and Group Discussions

Guided self-reflection and peer discussions, facilitated by the peer trainer, are embedded throughout the training to deepen understanding and foster mutual motivation. After the first part of the introductory lecture, GPs share positive and challenging experiences with PA advice, including personal barriers such as their own PA levels or attitudes.

During the presentation of the 3As method in part 2 of the introductory lecture, discussions focus on conversation openers and strategies for assessing patients’ PA levels, with key points and good practice examples documented on a flipchart for later reference.

At the close of the training, participants reflect on how they will apply their learning in routine practice, guided by the following *Monday question*: “What will you change or do differently next Monday (or any other next working day) in your practice?” The peer trainer reinforces this by suggesting practical strategies (eg, adding notes to patient records and adapting workflows), with reflections again noted on a flipchart to visualize intended actions.

#### Moderated Role-Play Sessions With Peer Feedback

Three different CHD case vignettes, each describing the patient’s personal and medical background, were developed by the study team in collaboration with the Communication in Medical Education team at the Medical Faculty of Heinrich-Heine-University, a patient representative, and a GP. These role-plays allow GPs to practice very brief PA advice using the 3As method.

Each training includes 2-3 15-minute role-plays, with the number depending on the time required for participant questions, feedback, and discussion within the role-play block. A fourth role-play may be added if time permits. Each role-play is followed by structured peer feedback facilitated by the GP trainer. Feedback follows a set sequence: the participating GP, simulated patient, peer group, and trainers reflect in turn. Reflections focus on use of the 3As model, its influence on the consultation, and areas for improvement. The role-play session lasts approximately 105 minutes, including discussions and a flexible short break as needed.

#### Experiential Learning Through Direct Engagement With PA Stimuli

GPs reported a preference for interactive formats with PA elements [36]. Given time constraints and participants’ personal comfort, we avoided extended PA activities and instead integrated low-threshold PA stimuli throughout the training. Next to the break area, simple equipment (eg, resistance bands) and illustrated exercise cards encourage spontaneous engagement and ideas for patient discussions. An information table displays flyers on local PA programs and educational materials from reputable public health institutions (eg, German Federal Institute of Public Health). Trainers also encourage participants to stand and move during sessions, supported by standing tables. These low-threshold elements normalize PA as part of the training experience and provide GPs with resources to share with patients.

#### Practical Materials Offering PA Recommendations for GPs and Patients

Two handouts support PA advice. The GP-focused version summarizes the 3As method with example phrases, WHO-based PA recommendations, brief guideline-based guidance on PA in stable and unstable CHD based on the German national CHD guidelines [8], practical tips for promoting daily PA, and information on digital health applications.

The patient handout provides general PA recommendations, information on health insurance offers, information on digital health applications, links to exercise resources provided by health organizations, and contacts for local rehabilitation and cardiac sports groups. The patient-facing materials have not yet undergone formal readability or health-literacy testing or specific adaptation for patients with lower educational attainment. All handouts and training materials are available after the training through the password-protected digital OptiCor training portal (Multimedia Appendix 5) [46].

Each GP receives a coffee mug printed with an infographic summarizing the WHO PA recommendations, which serves as a tangible prompt in everyday practice and is coded as “prompts/cues” and “adding objects to the environment” in the BCT mapping.

#### Good Practice Videos Available On-Demand After the Session

Two brief demonstration videos showing standardized PA advice using the 3As method are available through the training portal to reinforce learning and support long-term retention (Multimedia Appendix 5). Viewing is optional and not routinely monitored. The videos remain accessible for the duration of the project, including for at least 6 months after the final study-related training session.

## Discussion

### Main Study Findings

Within the framework of a 5-year funded health services research program in German general practice, we systematically developed a 3.5-hour small-group training approach to support GPs in delivering very brief PA advice to patients with CHD. The formative process produced a refined target behavior, a COM-B/TDF-informed behavioral diagnosis, selected intervention functions, BCT-specified components, and a post-rehearsal training package. The training intervention combines theoretical input, reflection, role-play, and practical resources, and it is delivered by a GP peer trainer and scientific lead using a detailed manual. It targets knowledge, skills, perceived feasibility, confidence, and motivation identified in the formative work.

### Interpretation and Implications

We are not aware of any other systematically developed GP training in Germany that focuses on very brief PA advice using the 3As approach for patients with CHD. This gap is relevant because PA promotion in CHD requires a pragmatic approach that fits routine consultations while addressing disease-specific concerns and counseling barriers. Despite methodological parallels to our prior smoking cessation training [26], we did not simply adapt the program, as smoking cessation and PA promotion differ substantially in their behavioral determinants and counseling requirements due to broader motivational and contextual factors affecting PA in both patients (eg, fear of worsening symptoms) and GPs (eg, concerns about the patient-doctor relationship).

### Comparison With Prior Work

International training programs have been developed to support PA advice by HPs, targeting HP behavior [47,48], patient behavior [49], or both [50]. These vary in duration (1‐2 hours [49,51] to 2‐3 days [52,53]), content (eg, guidelines/PA recommendations [48], practical exercises to check physiological parameters [48], written PA prescriptions [47], and role-plays [49,54]), and delivery (in-person [47,51] or online [50,55]). Many draw on theories, such as the transtheoretical model [51,53,56], self-efficacy theory [56], and theory of planned behavior, or health literacy models, and include approaches like motivational interviewing [47,52,53] and the 5As model [49,51]. However, few interventions address the specific needs of HPs when providing PA advice to defined patient groups (eg, those with diabetes [50,56]). Even fewer report a systematic intervention development process [50]. Our training adds a time-efficient, theory-informed intervention tailored to delivering PA advice in routine general practice in Germany and integrates empirical findings, guideline recommendations, and GP and patient input.

### Equity, PA-Safety Concerns, and Transferability

Equity was considered because prior survey findings indicated that women, younger individuals, those with lower education, and physically inactive individuals were more likely to report no GP advice on PA [21]. The training emphasizes routine, nonselective PA advice irrespective of patient characteristics, assumed motivation, education, gender, or the GP’s own PA habits. In line with our broader communication-training approach, patient-centered communication, shared decision-making, and nonstigmatizing language are addressed as underlying principles throughout the training (eg, through reflective and careful trainer feedback following role-plays), although they were not developed as separate equity-specific components. Whether the 3As-based training supports more consistent advising will be examined in the ongoing full-scale pragmatic cRCT, including subgroup analyses where feasible [24]. Concerns about PA safety in CHD were identified as potential barriers and were discussed with experienced GP trainers during development. Following their advice, safety was initially addressed cautiously rather than as a separate module, mainly through guideline-based PA recommendations and discussion of patients’ worries about overexertion, to avoid unintentionally amplifying hesitancy. The subsequent pilot cRCT and its accompanying process evaluation suggested that GPs’ own safety concerns may need to be addressed somewhat more explicitly; this pilot-informed refinement is reported separately [28].

The training appears broadly transferable within Germany, as its 3As structure, guideline-based PA and CHD content, peer-trainer approach, case vignettes, role-play and feedback structure, generic handout content, digital training portal, and BCT-linked logic are not region-specific. Adaptations would mainly concern information/materials on local referral options, rehabilitation or cardiac sports groups, and health insurance offers. International transfer also appears feasible because the core principles are not specific to the German health care system, although adaptation to national care pathways, reimbursement structures, professional roles, and available PA support services would be required.

### Implications for Future Research

Following the formative development reported here, the training and evaluation procedures were piloted under real-world conditions in a pragmatic pilot cRCT, which has been reported separately [28]. Findings from this pilot informed subsequent refinements to the training version and procedures used in the ongoing full-scale pragmatic cRCT, which examines effects on GPs’ PA advising behavior and is accompanied by a process evaluation. Future analyses should assess whether the training increases not only the frequency of PA advice, but also the completeness of the 3As method and the consistency of advice across patient groups.

Future adaptations for other chronic conditions, settings, or digital formats are conceivable. However, consistent with the behavioral theory, such adaptations may require renewed behavioral diagnosis and context-specific tailoring.

### Strengths and Limitations

A key strength is the integration of extensive prior research with 49 GPs [22,36] and 33 patients [37], informing the specification of the target behavior, barriers, and training needs. The systematic development process explicitly linked behavioral determinants, intervention functions, BCTs, and training components, thereby making transparent how empirical findings, behavior change theory, clinical recommendations, and GP and patient input shaped the final intervention. The detailed intervention description using the TIDieR checklist [34] supports consistent implementation, evaluation, replication, and future adaptation.

At the time of intervention development, not all underlying formative sources had completed peer review, which should be considered when interpreting the formative evidence base. However, the survey and GP studies are now available as peer-reviewed publications [21,22,36], while the patient-perspective study is cited transparently as a conference output [37]. A limitation is that while we systematically mapped training components to BCTs, their inclusion alone does not guarantee their effectiveness. As Michie et al [57] point out, BCTs are precise enough to achieve reliability of recognition but not inherently indicative of effectiveness. In contrast, in the intervention mapping framework, behavior change methods are assumed to be effective if key parameters for use are met during their practical application [58]. To support the correct application of BCTs when delivering the training, we applied several strategies, including interdisciplinary discussion of BCTs, standardization through a detailed trainer manual, a preimplementation training rehearsal, trainer briefings, refresher sessions, and on-site implementation support.

A further limitation is that our qualitative interview and focus group guides for the underlying needs assessment were not originally developed based on the COM-B framework or TDF. We will address this in the process evaluation conducted alongside the ongoing full-scale pragmatic cRCT by aligning interview guides and coding with these frameworks. We also expect to gather indications regarding the delivery of core components and how intended BCTs were implemented in practice. Some development decisions, including the pragmatic use of APEASE, relied on multidisciplinary team judgment rather than formal scoring. This reflects the applied nature of developing a feasible training for routine general practice.

### Conclusions

This rigorously developed intervention is designed to support GPs in providing very brief PA advice to patients with CHD. Developed through a transparent, systematic process and informed by empirical findings, behavior change theory, clinical recommendations, and GP and patient input, the intervention is tailored to GPs’ needs and routine general practice. Following the formative development reported here, the training and evaluation procedures were piloted under real-world conditions, and the intervention is currently being evaluated in an ongoing full-scale pragmatic cRCT. While its effectiveness remains to be established, the transparent and theory-linked development approach may also inform the adaptation of brief behavior change training to other patient groups, settings, and delivery formats.

## Supplementary material

10.2196/89467 Multimedia Appendix 1 Exemplary contents of the developed general practitioner training with corresponding behaviour change technique codes and labels.

10.2196/89467 Multimedia Appendix 2 Table of contents of the trainer manual.

10.2196/89467 Multimedia Appendix 3 Training agenda.

10.2196/89467 Multimedia Appendix 4 Outline of the introductory lecture.

10.2196/89467 Multimedia Appendix 5 Screenshots of the training portal and videos.

10.2196/89467 Checklist 1 TIDieR checklist.

## Abbreviations

APEASE: affordability, practicability, effectiveness and cost-effectiveness, acceptability, side effects/safety, and equity
BCT: behavior change technique
BCW: behavior change wheel
CHD: coronary heart disease
CME: continuing medical education
COM-B: capability, opportunity, motivation-behavior
cRCT: cluster randomized controlled trial
GP: general practitioner
HP: health professional
PA: physical activity
TDF: theoretical domains framework
TIDieR: Template for Intervention Description and Replication
WHO: World Health Organization
