# Supplementary material for: Systematic Development of a Training Intervention to Support General Practitioners’ Very Brief Physical Activity Advice for Patients With Coronary Heart Disease: Formative Intervention Development Study From the OptiCor Project
Source: JMIR Form Res. 2026 Oct 22;10:e89467. doi: 10.2196/89467 (PMC13717261; doi:10.2196/89467)
Supplement: Supplementary file 1 [file formative-v10-e89467-s001.docx]

**Multimedia Appendix 1. Exemplary contents of the developed general practitioner training with corresponding behaviour change technique codes and labels [1].**

| **BCT group** | **BCT code and label** | **Examples of implementation within the training** |
| --- | --- | --- |
| **1. Core component of the training: Introductory lecture for theoretical input** | | |
| **4. Shaping knowledge** | 4.1 Instruction on how to perform a behaviour | Provide information about very brief PA advice according to 3As. |
| **5. Natural consequences** | 5.1 Information about health consequences | Provide evidence on the effectiveness of HPs’ very brief advice on patients’ PA, provide evidence on health risks of inactivity in CHD and benefits of PA. |
|  | 5.2 Salience of consequences | Provide information on consequences of PA or inactivity by using a demonstration of a cardiovascular risk calculator/score. |
|  | 5.3 Information about social and environmental consequences | Provide information about cost-effectiveness of PA advice and options for reimbursement of providing PA advice in general practice (note: it is not reimbursed directly). |
|  | 5.6 Information about emotional consequences | Clarify that providing very brief PA advice can lead to short-term rather than long-term success helping to manage GPs’ expectations and reduce frustration, and ultimately enhance self-efficacy.  Explain how applying the 3As method to deliver PA advice can reduce stress and frustration in daily GP practice, leading to increased satisfaction. |
| **6. Comparison of behaviour** | 6.1 Demonstration of the behaviour | Provide information on how to start a conversation on PA with patients by giving examples (pictures/sentences). |
|  | 6.3 Information about others’ approval | Provide information* that patients with CHD are more likely to appreciate a supportive conversation about PA, focusing on assistance rather than criticism or reproach, which could lead to feelings of guilt. |
| **8. Repetition and substitution** | 8.2 Behaviour substitution | Suggest that GPs should focus on providing support with PA to patients (3As: assist) rather than focussing on time-consuming discussions on patients’ PA motivation. Ideally by asking the patient what they would prefer. |
| **9. Comparison of outcomes** | 9.1 Credible source | Provide recent evidence-based data on the importance and effectiveness of very brief GP PA advice. |
| **11. Regulation** | 11.2 Reduce negative emotions | Provide strategies for GPs on how to reduce frustration during PA advice (e.g., through realistic goal setting). |
| **13. Identity** | 13.2 Framing/reframing | Provide information that medical advice on PA must not necessarily be time consuming and frustrating/exhausting (cognitive structuring). |
| **15. Self-belief** | 15.1 Verbal persuasion about capability | Inform GPs that patients perceive them as a trusted source of health information, and that they can successfully advise on PA even if they are not personally active themselves—addressing self-doubts and reinforcing their ability to succeed. |
| **2. Core component of the training: Guided self-reflection sessions and group discussions for in-depth exploration of personal practice and barriers** | | |
| **1. Goals and planning** | 1.1 Goal setting (behaviour) | Encourage GPs to set a self-defined goal for the upcoming working day regarding the provision of PA advice by using the “Monday Question”: *“What would you change or do differently next Monday (or next working day) in your practice?”* This reflective question helps GPs focus on continuous improvement and ensures they are consistently integrating PA discussions into their patient interactions. |
|  | 1.2 Problem solving | Encourage GPs to identify barriers that may prevent them from providing PA advice and discuss potential strategies to overcome these challenges. Additionally, encourage GPs to recognise facilitators and specific triggers that help promote the integration of PA advice into their practice, and use these insights to develop a personalised strategy to consistently motivate themselves to engage in these important conversations with patients. |
|  | 1.4 Action planning | Encourage GPs to actively plan their delivery of PA advice to strengthen their implementation intentions. This can include considering the context in which they will provide advice (e.g., during routine check-ups), the frequency with which they will integrate it into consultations, and specific strategies to ensure consistent implementation. By making concrete plans, GPs are more likely to follow through and effectively incorporate PA advice into their daily practice. |
| **3. Social support** | 3.2. Social support (practical) | Provide practical support for delivering PA advice by offering guidance on addressing common barriers. This support comes from peer trainers and participating GPs, who can share effective strategies, real-world examples, and good practices. Facilitating peer discussions can help GPs feel more confident and equipped to integrate PA advice into their routine consultations. |
|  | 3.3. Social support (emotional) | Provide emotional support for GPs in delivering PA advice by creating opportunities for peer trainers and participating GPs to share experiences, challenges, and coping strategies. This can include discussing ways to handle frustration, patient resistance, or time constraints, fostering a supportive environment where GPs feel encouraged and motivated to continue integrating PA advice into their practice. |
| **5. Natural consequences** | 5.4. Monitoring of emotional consequences | Encourage GPs to reflect on and openly express their feelings regarding the provision of PA advice. Creating a space for self-reflection and discussion can help GPs identify emotional barriers, recognise successes, and develop strategies to enhance their confidence and motivation in delivering PA advice effectively. |
| **6. Comparison of behaviour** | 6.2. Social comparison | Encourage GPs to compare their own experiences and behaviours in providing PA advice with those of their colleagues during past routine consultations. This reflective process can help identify best practices, uncover common challenges, and inspire new strategies for more effective and confident PA advice. |
| **9. Comparison of outcomes** | 9.2 Pros and cons | Encourage GPs to reflect on the advantages and potential challenges of providing PA advice more frequently to their patients with CHD. This self-reflection can help them recognise the positive impact of PA advice – such as improved patient health outcomes and enhanced patient-physician relationships – while also acknowledging potential barriers, such as time constraints or patient resistance. |
| **13. Identity** | 13.3 Incompatible beliefs | Encourage GPs to reflect on gaps between their PA advice practices and their self-image as health consultants, motivating them to align their actions with their professional goals. |
| **15. Self-belief** | 15.3 Focus on past success | Encourage GPs to reflect on past strategies that led to successful PA conversations with CHD patients. What worked well? Identifying effective approaches can help refine future consultations. |
| **3. Core component of the training: Moderated role-play sessions with peer feedback (practice element)** | | |
| **1.Goals and planning** | 1.1. Goal setting (behaviour) | Encourage GPs to apply all elements of the 3As method during the role-play. |
|  | 1.2. Problem solving | Encourage GPs to identify barriers preventing them from applying the 3As method (or specific elements of this method) in discussions following each role-play. |
|  | 1.5. Review behaviour goal(s) | Examine how well a GP’s performance during role-play corresponds to the agreed goal of applying all elements of the 3As method and consider modifying the behavioural goal, e.g., through realistic goal setting. |
|  | 1.6. Discrepancy between current behaviour | Encourage GPs to reflect on which elements of the 3As method they applied and which they did not. Emphasise the message “every step / every minute counts” and highlight everyday activities over a narrow focus on meeting the 150–300 minutes/week target or promoting only “sport.” GPs are encouraged to try out new ways of engaging patients – for example, by asking open questions or using the example phrases from the 3As approach. |
| **2. Feedback and monitoring** | 2.2. Feedback on behaviour | Provide moderated feedback on GP’s performance during role-play by peer trainers and participating GPs. |
|  | 2.7. Feedback on outcome(s) of behaviour | Provide moderated feedback on outcomes (e.g., the simulated patients’ reaction) by peer trainers, participating GPs, and simulated patients (actors) themselves. |
| **3. Social support** | 3.2. Social support (practical) | Provide practical support (e.g., advice on how to cope with a specific patient reaction) by peer trainers and participating GPs. |
|  | 3.3. Social support (emotional) | Ensure that emotional support is available through peer trainers and participating GPs. |
| **4. Shaping knowledge** | 4.1. Instruction on how to perform behaviour | Reinforce learning by repeatedly providing verbal instructions on delivering very brief PA advice using the 3As method. |
| **5. Natural consequences** | 5.4. Monitoring of emotional consequences | Encourage GPs to reflect and reveal their feelings after the role-play, particularly with regard to a reduction of negative feelings. |
| **6. Comparison of behaviour** | 6.1. Demonstration of the behaviour | Provide role-plays with moderated peer feedback to practice the delivery of very brief advice on PA according to the 3As method. |
|  | 6.2. Social comparison | Encourage GPs to observe the advising behaviour of colleagues during role-play allowing internal comparisons with their own behaviour during role-play but also during their previous routine practice consultations. |
|  | 6.3. Information about others’ approval | Provide feedback on the advising behaviour of the GP who participates in the role-play given by peer trainers and participating GPs. |
| **8. Repetition and substitution** | 8.1. Behavioural practice/rehearsal | Provide role-plays with moderated feedback to practise the delivery of very brief GP advice on PA. |
|  | 8.2. Behavioural substitution | Provide alternative reactions/communication styles and content/ recommendations after role-plays by peer trainers and participating GPs. |
|  | 8.7. Graded tasks | Provide role-plays with moderated feedback to practise the delivery of very brief GP advice on PA with different CHD patient profiles, varying in PA levels and experience, personality, anxiety, and PA motivation.. |
| **9. Comparison of outcomes** | 9.1. Credible sources | Facilitate role-play experiences that allow GPs to observe both effective communication and areas for improvement in consultations conducted by participating GPs. |
| **11. Regulation** | 11.2. Reduce negative emotions | Use role-plays to illustrate how very brief PA advice can be seamlessly integrated into routine practice, showing that it does not necessarily lead to frustration or prolonged discussions with patients. |
| **15. Self-belief** | 15.3. Focus on past success | Follow each role-play with a discussion, inviting the participating GP to reflect on what worked well and share their insights. |
| **4. Core component of the training: Experiential learning through direct engagement using PA stimuli (self-experience)** | | |
| **4. Shaping knowledge** | 4.1 Instruction on how to perform behaviour | Provide information and hands-on opportunities for GPs to engage with PA by displaying simple training equipment (e.g., resistance bands) and exercise instruction cards. |
| **6. Comparison of behaviour** | 6.1 Demonstration of the behaviour |  |
| **7. Associations** | 7.1. Prompts/cues | Memorable element of the training to remember to provide advice on PA. Displaying this alongside key messages about the benefits of PA can reinforce the importance of consistently providing PA advice during patient consultations. |
| **11. Regulation** | 11.2. Reduce negative emotions | Encourage GPs to practice simple exercises to challenge beliefs that PA must be time-consuming or require equipment, making it easier to promote accessible PA to patients. |
| **13. Identity** | 13.2 Framing/Reframing |  |
| **5. Core component of the training: Practical materials offering PA opportunities and recommendations for both GPs and patients** | | |
| **4. Shaping knowledge** | 4.1 Instruction on how to perform a behaviour | Provide leaflets for GP practice rooms containing information on delivering PA advice, including the 3As method, evidence-based PA information, and PA conversation openers to facilitate effective communication with patients. |
| **7. Associations** | 7.1 Prompts/cues | Provide leaflets for GP practice rooms as reminders for delivering PA advice, including the 3As method, evidence-based PA information, and details on regional PA programs to help GPs in offering relevant and practical recommendations to patients. |
| **11. Regulation** | 11.3 Conserving mental resources | Encourage GPs to utilise the compiled information on regional PA programs to minimise the use of their own mental resources, allowing them to focus more on effective communication with patients with CHD. |
| **12. Antecedents** | 12.5 Adding objects to the environment | Encourage GPs to keep leaflets—both for their personal reference and for forwarding to patients—within easy reach or on their desks. These leaflets include the 3As method, evidence-based PA information, and details on regional PA programs, ensuring GPs have quick access during conversations with patients who have coronary heart disease (CHD).  Additionally, provide a small gift, such as a coffee cup featuring national PA recommendations, as a thoughtful reminder for GPs to discuss PA with patients during practice consultations. |
| **6. Core component of the training: Good practice videos available on-demand following the training** | | |
| **6. Comparison of behaviour** | 6.1. Demonstration of the behaviour | Provide good-practice-videos demonstrating the delivery of very brief GP advice on PA based on the 3As method. |
| **11. Regulation** | 11.2. Reduce negative emotions | Provide good practice videos demonstrating that GP advice on PA can be seamlessly integrated into routine practice, and that it does not necessarily lead to frustration or conflict with the patient. |
| **13. Identity** | 13.2. Framing/reframing |  |
| **16. Covert learning** | 16.3. Vicarious consequences |  |

Abbreviations: BCT, behaviour change technique; CHD, coronary heart disease; GP, general practitioner; HP, health professional; PA, physical activity; WHO, World Health Organization.

***Based on current literature and our comprehensive qualitative and quantitative needs assessment in GPs and patients with CHD [2-5].

**References**

1.	Michie S, Richardson M, Johnston M, Abraham C, Francis J, Hardeman W, et al. The behavior change technique taxonomy (v1) of 93 hierarchically clustered techniques: building an international consensus for the reporting of behavior change interventions. Ann Behav Med. 2013 Aug;46(1):81-95. PMID: 23512568. doi: 10.1007/s12160-013-9486-6.

2.	Prinz A, Hoppe S, Leve V, Crutzen R, Donner-Banzhoff N, Wilm S, et al. Experiences, perceptions and attitudes on providing advice on physical activity to patients with chronic ischaemic heart disease: a qualitative study in general practitioners in Germany. Fam Med Community Health. 2025;5(13(3)):e003124. doi: 10.1136/fmch-2024-003124.

3.	Prinz A, Hoppe S, Crutzen R, Wilm S, Kastaun S. General practitioners' educational and training needs and requirements for advising patients with coronary heart disease on physical activity: findings from a qualitative study in Germany. BMC Prim Care. 2025 Aug 29;26(1):273. PMID: 40883666. doi: 10.1186/s12875-025-02973-0.

4.	Hoppe S, Prinz A, Kotz D, Mons U, Kuss O, Crutzen R, et al. Physical activity advice from general practitioners in Germany: findings from a cross-sectional population survey of individuals with chronic ischaemic heart disease (OptiCor study). BMJ open. 2026 Feb 23;16(2):e101017. PMID: 41730561. doi: 10.1136/bmjopen-2025-101017.

5.	Hoppe S, Prinz A, Crutzen R, Donner-Banzhoff N, Icks A, Kotz D, et al. Optimising the treatment of chronic ischemic heart disease by training general practitioners to deliver very brief advice on physical activity (OptiCor): protocol of the systematic development and evaluation of a complex intervention. BMC Prim Care. 2024 Nov 27;25(1):404. PMID: 39604870. doi: 10.1186/s12875-024-02655-3.
