# Supplementary material for: Systematic Development of a Training Intervention to Support General Practitioners’ Very Brief Physical Activity Advice for Patients With Coronary Heart Disease: Formative Intervention Development Study From the OptiCor Project
Source: JMIR Form Res. 2026 Oct 22;10:e89467. doi: 10.2196/89467 (PMC13717261; doi:10.2196/89467)
Supplement: Supplementary file 2 [file formative-v10-e89467-s002.docx]

[www.opticor-study.info](https://www.opticor-study.info/)

Additional file 2, Version 1-1 (translated from German into English)

28.07.25

This work is licensed under the Creative Commons Attribution

**Table of contents of the trainer manual**

**1.	Training Preparation (OptiCor Study Team)**

1.1	Training Materials

1.1.1	Before the Training

1.1.2	During the Training

1.2	Seminar Room Setup

**2.	Programme Schedule (Trainer Edition)**

**3.	Training Introduction**

3.1	Welcome

3.2	Ground Rules

3.3	Participant Introductions: Expectations and Goals

3.4	Training Objectives in the Study Context

3.5	Programme Overview

**4.	Impulse Lecture – Part 1**

4.1	Coronary Heart Disease (CHD) – Prevalence, Morbidity, and Mortality

4.2	Physical Activity (PA) and CHD

4.2.1	WHO Definitions Related to PA

4.2.2	Effects of PA on CHD

4.2.3	General PA Recommendations

4.2.4	Additional PA Recommendations

4.2.5	The “10,000 Steps” Myth?

4.2.6	Activity Levels among Individuals with CHD

4.3	National Treatment Guideline “Chronic CHD” – Key Recommendations

4.3.1 Recommendations for Measures According to Risk Classes

4.3.2 PA Recommendations by Risk Class

4.3.3 Recommendations on Exercise Intensity

4.4	PA and Other Conditions

**5.	Experience Exchange/Discussion**

5.1 Group Discussion and Sharing Experiences

5.2 Barriers Encountered by General Practitioners (GPs)

5.3 GP Practice as a Key Setting for Brief Advice on PA

**6.	Impulse Lecture – Part 2**

6.1	Effectiveness of Brief Advice on PA

6.2	Definition of Brief Advice on PA according to NICE^[1]^

6.3	Brief Advice Methods

**7.	The 3As Method**

7.1	Before Applying the Method

7.2	Ask: Assessing the Level of PA

7.3	Advise: Encouraging Increased or Regular Activity

7.4	Assist: Providing Support and Specific Recommendations

**8.	Scheduled Break**

**9.	Case-Based Practical Exercises in Small Groups – Part 1 & 2**

9.1 Role-Play Didactics: Detailed Instructions

9.1.1	Group Size

9.1.2	Exercise Procedure (Small Groups)

9.1.3	Trainer’s Role (Role-Play Facilitation)

9.1.4	Feedback Rules for Participants and Trainers

9.1.5	Role-Play Instructions

9.1.6	Conducting the Exercise

9.1.7	Timeline for the Exercise

9.2.	Trainer Techniques During the Role-Plays

9.2.1.	The Feedback Round

9.2.2.	The “Time-Out” Rule

9.3.	Debriefing the Exercises

**10.	Reflection**

10.1	“Monday Question”

10.2	Key Take Home Messages (Optional)

**11.	Closing and Farewell**

**12.	Handling Challenging Situations**

12.1	Managing Resistance During Training

12.2	Sensitive Topics in the Context of GP PA Advice on

**13.	A Training on PA Without Any Activity?**

**14.	Warm-Up Exercise Didactics – Use Only If Necessary (Time-Dependent!)**

**15.	References**

**Additional Material**

- Handouts for GPs and Patients
- Case Vignettes for Role-Plays and Character Profiles for Simulated Patients

1. Campbell F, Blank L, Messina J, Day M, Woods HB, Payne N, et al. Physical activity: Brief advice for adults in primary care. Public Health Intervention Guidance. National Institute for Health and Clinical Excellence; 2012.
