# Supplementary material for: Systematic Development of a Training Intervention to Support General Practitioners’ Very Brief Physical Activity Advice for Patients With Coronary Heart Disease: Formative Intervention Development Study From the OptiCor Project
Source: JMIR Form Res. 2026 Oct 22;10:e89467. doi: 10.2196/89467 (PMC13717261; doi:10.2196/89467)
Supplement: Supplementary file 3 [file formative-v10-e89467-s003.docx]

[www.opticor-study.info](http://www.opticor-study.info)

Additional file 3, Version 1-1 (translated from German into English)

This work is licensed under the Creative Commons Attribution

28.07.25

**Training agenda**

*Schedule applies to weekday sessions, e.g., Wednesday or Friday. Timings may vary for weekend trainings.*

**15:30 – 15:40	Introduction** (Scientific team member and peer trainer)

*Welcome, participants introductions and their expectations, training objectives, programme introduction*

**15:40 – 15:55	Introductory Lecture – Part 1** (Scientific team member)

*Epidemiology of Coronary Heart Disease (CHD), risk factors, definition of physical activity (PA), PA & CHD, guideline recommendations, myth “10.000 steps”, activity levels of people with CHD, recommendations of the German treatment guideline “chronic CHD”, PA and other diseases*

**15:55 – 16:05	Experiences Exchange & Discussion** (Peer trainer)

*Topic: PA advice in general practice – successes, barriers, and opportunities.*

*Following the discussion: Findings on GP-experienced barriers (OptiCor qualitative study results) and discussion of general practice as a key setting for PA counselling.*

**16:05 – 16:15	Introductory Lecture – Part 2** (Scientific team member)
*Effectiveness of PA advice, effectiveness of brief PA advice*

**16:15 – 16:30	The 3As Method** (Scientific team member)

Introduction of the method, *Ask/Assess: Assessing PA level (incl. peer exchange), Advise: Advice on PA, Assist: Provision of support (e.g., recommendations)*

**16:30 – 16:45	Break** (Sandwiches, coffee, tea, water, juice)

**16:45 – 18:30	Small Group Practical Exercises** (Peer trainer)

*Introduction to feedback rules, 2-3 role plays with simulated patients, moderated feedback and discussion, clarification of questions*

**18:30 – 18:45	Reflection** (Scientific team member)

*“What will I do differently in practice on Monday”?* Open questions and review of collected learning goals (flip chart).

**18:45 – 19:00	Closing & Farewell** (Scientific team member and peer trainer)

*Completion of evaluation forms, distribution of certificates of participation, and thank-you gifts (training mugs)*
