# Supplementary material for: Systematic Development of a Training Intervention to Support General Practitioners’ Very Brief Physical Activity Advice for Patients With Coronary Heart Disease: Formative Intervention Development Study From the OptiCor Project
Source: JMIR Form Res. 2026 Oct 22;10:e89467. doi: 10.2196/89467 (PMC13717261; doi:10.2196/89467)
Supplement: Supplementary file 4 [file formative-v10-e89467-s004.docx]

[www.opticor-study.info](https://www.opticor-study.info/)

Additional file 4, Version 1-1 (translated from German into English)

28.07.25

This work is licensed under the Creative Commons Attribution

**Outline of the introductory lecture**

**Coronary Heart Disease (CHD) *–* Prevalence, Morbidity & Mortality**

- **Epidemiological Overview:** Prevalence, morbidity, and mortality rates of CHD
- **Health and Economic Impact:** Consequences for individuals and healthcare systems
- **Socio-demographic Risk Factors:** Influence of age, gender, and socioeconomic status
- **Lifetime Risk:** Differences in lifetime risk based on gender and social background
- **Behavioural Risk Factors:** Key lifestyle contributors to CHD onset and progression

**Physical activity (PA) and CHD** **– Recommendations & Relevance**

- **Secondary Prevention:** Evidence for PA as an effective preventive strategy in the management of CHD (systematic review)
- **Impact of PA:** Effects of regular PA on myocardial infarction risk, cardiovascular mortality, and hospitalisation
- **Comparison with Medication:** Illustration of PA’s impact versus statin therapy using a cardiovascular risk calculator (ARRIBA^[1]^).
- **WHO Guidelines:** Current recommendations for weekly PA.
- **The “10,000 Steps” Myth**: Evidence-based clarification based on a recent meta-analysis.
- **Activity Levels in CHD Patients:** Patterns of PA behaviour among individuals with CHD

**German treatment guideline ’Chronic Coronary Heart Disease’: Recommendations on PA and Advice on PA**

- **Key Recommendations:** Summary of national guideline advice on PA in CHD management.
- **Cross-Disease Relevance:** Information that recommendation to advice on PA is also relevant for the management (and thus mentioned in the respective treatment guidelines) of other chronic conditions (e.g., diabetes, depression)

**Experiences of PA Advice in GP practice**

- **Experienced Barriers:** Literature and OptiCor findings on GPs’ barriers to PA counselling.
- **Primary Care as a Key Setting**: General practice as an ideal, low-threshold environment for initiating brief PA interventions

**Effectiveness of PA Advice in the healthcare setting**: Evidence and information on the added value of brief interventions (systematic reviews and meta-analyses)

**Implementing Brief Advice on PA in general practice: The 3As Method**

- **The 3As Method:** Introduction of the 3As method
- **Ask /Assess:** How to ask for patient’s PA
- **Advise:** How to advise patients with CHD on PA
- **Assist:** How to offer practical and patient-centred support, and information on concrete resources (e.g., digital health tools, exercise groups, cardiac rehab, reliable information)
- **Address Concerns:** Respond empathetically to questions, doubts, and perceived barriers to behaviour change

1. Angelow A, Klötzer C, Donner-Banzhoff N, Haasenritter J, Schmidt CO, Dörr M, et al. Validation of cardiovascular risk prediction using the arriba instrument. [Validierung der kardiovaskulären Risikoprädiktion des arriba-Instruments}. Dtsch Arztebl International. 2022;119(27–28):476–82.
