# Supplementary material for: Systematic Development of a Training Intervention to Support General Practitioners’ Very Brief Physical Activity Advice for Patients With Coronary Heart Disease: Formative Intervention Development Study From the OptiCor Project
Source: JMIR Form Res. 2026 Oct 22;10:e89467. doi: 10.2196/89467 (PMC13717261; doi:10.2196/89467)
Supplement: Supplementary file 5 [file formative-v10-e89467-s005.docx]

[www.opticor-study.info](https://www.opticor-study.info/)

Additional file 5, Version 1-1

28.07.25

This work is licensed under the Creative Commons Attribution

**Screenshots of Training Portal and Videos**

**Access via the OptiCor website (content in German)**

**Overview of Training Portal Resources**

Includes downloadable materials: the introductory lecture (theoretical foundations) and practical resources for GPs (e.g., a handout with an overview of the 3As method and PA recommendations) as well as resources for their patients (e.g., contact information for local rehabilitation or cardiac sports groups).

**“Good Practice” Videos:** Brief PA advice for CHD patients using the 3As method, demonstrated with an anxious and an unmotivated patient.
